# Supplementary material for: Probiotics reshape the coral microbiome in situ without detectable off-target effects in the surrounding environment
Source: Commun Biol. 2024 Apr 9;7:434. doi: 10.1038/s42003-024-06135-3 (PMC11004148; doi:10.1038/s42003-024-06135-3)
Supplement: Supplementary file 2 — Supplementary Information [file 42003_2024_6135_MOESM2_ESM.pdf]

Supplementary information of the Manuscript "Probiotics reshape the coral microbiome *in situ* without detectable off-targeted effects in the surrounding environment"

Authors: Nathalia Delgadillo-Ordoñez [1,2], Neus Garcias-Bonet [1], Inês Raimundo [1,2], Francisca C. García 1, Helena Villela [1], Eslam O. Osman [1], Erika P Santoro [1], Joao Curdia [1], Joao G D Rosado [1,2], Pedro Cardoso [1,2], Ahmed Alsaggaf [1,2], Adam Barno [1,2], Chakkiath Paul Antony [1], Carolina Bocanegra [1], Michael L. Berumen [1,2], Christian R Voolstra [3], Francesca Benzoni 1,2, Susana Carvalho [1,2], Raquel S Peixoto [1,2]

[1] King Abdullah University of Science and Technology (KAUST), Red Sea Research Center, Thuwal, 23955, Saudi Arabia

[2] Marine Science and Bioscience Programs, Biological and Environmental Science and Engineering Division, King Abdullah University of Science and Technology, Thuwal, 23955, Saudi Arabia

[3] Department of Biology, University of Konstanz, Konstanz, Germany

Supplementary Figures:

Supplementary Figure 1. Bacterial community structure of *Pocillopora verrucosa* according to sampling time and treatment. Nonmetric multidimensional scaling ordination (nMDS) of bacterial communities of coral in T1 (squares), T2 (circles), T3 (tringles), and T4 (trapeziums) (k = 2). The placebo (blue) and probiotic (pink) treatments are shown.

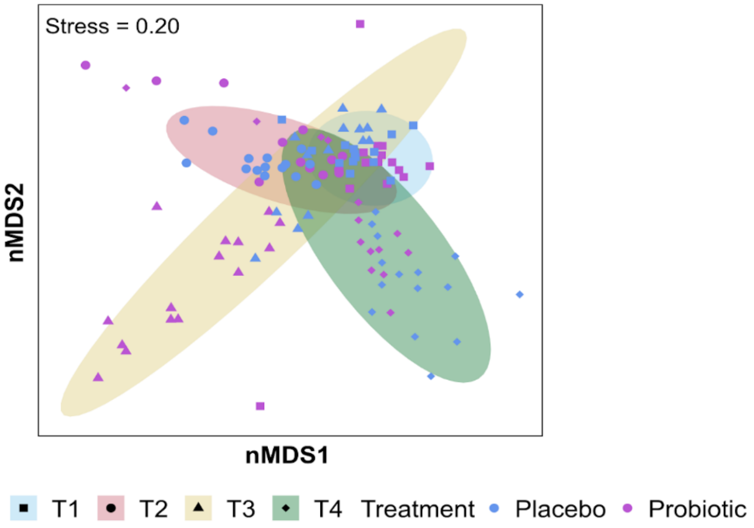

Supplementary Figure 2. Bacterial community structure of coral, seawater and sediment biological components. Nonmetric multidimensional scaling ordination (nMDS) of coral (circles), seawater (squares) and sediment (trapeziums) (k = 2). The placebo (blue) and probiotic (pink) treatments are shown.

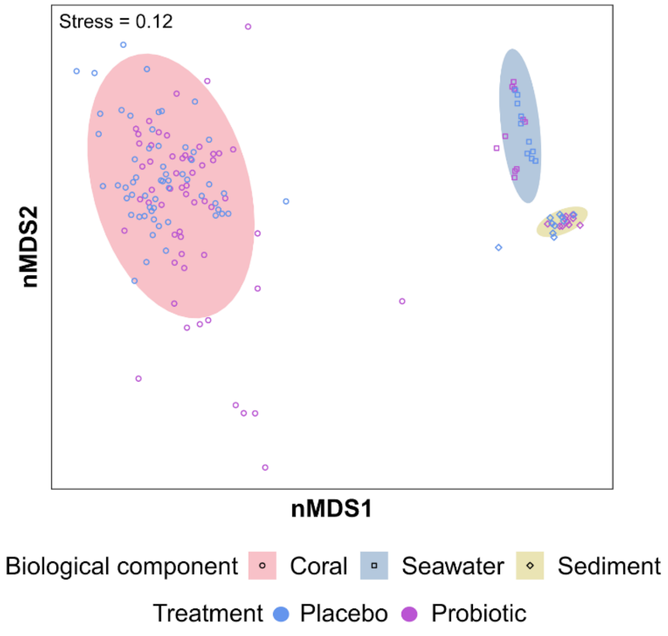

Supplementary Figure 3. *In situ* inoculation of the probiotic consortium to healthy colonies of *Pocillopora verrucosa* in the Coral Probiotic Village (CPV) in the Central Red Sea. 30 mL of the probiotic solution were released over the colony using a plastic syringe. Photo by Morgan Bennett-Smith.

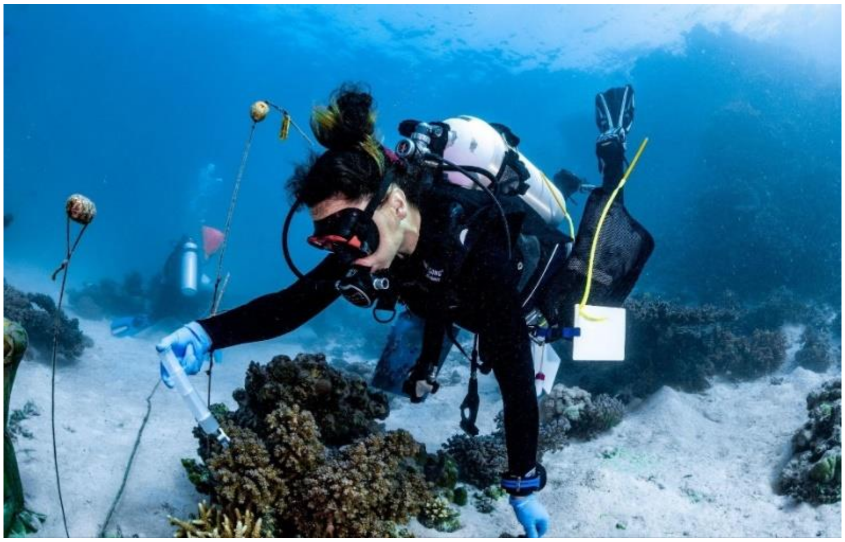

Supplementary Tables:

Supplementary Table 1. Summary of the BMC traits results for the six selected BMCs for the probiotic consortium. (-) indicates a negative result, (+) indicates positive result. For the catalase test, results are denoted with (+), (++), and (+++) from less to high production of bubbles, as a proxy of the catalase reaction.

**Strain**	**BMC ID**	**Coral-Host**	**Antagonism against *Vibrio coralliilyticus***	**Catalase**	**Siderophores production**	**Urease**	**Phosphate assimilation**
30H	*Pseudoalteromonas galatheae*	*Pocillopora verrucosa*	(-)	(+)	(-)	(-)	(-)
33H	*Pseudoalteromonas galatheae*	*Pocillopora verrucosa*	(-)	(+)	(-)	(-)	(-)
65H	*Cobetia amphilecti*	*Pocillopora verrucosa*	(-)	(+++)	(-)	(-)	(+)
81H	*Cobetia amphilecti*	*Pocillopora verrucosa*	(-)	(+++)	(-)	(-)	(+)
SAT10	*Halomonas* sp.	*Stylophora pistillata* Clade IV	(-)	(+++)	(+)	(-)	(-)
SAA19	*Sutcliffiella* sp.	*Galaxea fascicularis*	(-)	(+)	(+)	(-)	(-)

Supplementary Table 2. Normality test and comparisons between treatments (Placebo and Probiotic) of the top 10 most abundant families in T3.

**Normality test Shapiro Wilks**	***Burkholderiaceae***	***Endozoicomonadaceae***	***Lachnospiraceae***	***Phycisphaeraceae***	***Prevotellaceae***	***Rhodobacteraceae***	***Rikenellaceae***	***Simkaniaceae***	***Spirochaetaceae***	***Unclassified Alphaproteobacteria***
p-value	1.85E-10	0.01963	2.62E-08	1.46E-07	2.26E-09	1.39E-09	3.53E-09	4.93E-06	9.17E-07	7.26E-08
W	0.32762	0.91238	0.54874	0.61261	0.44659	0.42485	0.46633	0.72515	0.67424	0.5874
Interpretation	**Not normal**	**Not normal**	**Not normal**	**Not normal**	**Not normal**	**Not normal**	**Not normal**	**Not normal**	**Not normal**	**Not normal**
**Homogeneity of variances Levene test**										
Pr(>F)	0.5717	**0.03718**	**0.02292**	0.1128	**0.04166**	0.3982	**0.04562**	0.9164	0.717	0.8242
F-value	0.3278	4.8048	5.8191	2.6866	4.5742	0.737	4.3918	0.0112	0.1342	0.0503
Interpretation	**Homogeneity**	**Not homogeneity**	**Not homogeneity**	**Homogeneity**	**Not homogeneity**	**Homogeneity**	**Not homogeneity**	**Homogeneity**	**Homogeneity**	**Homogeneity**

**Wilcox test for comparisons between treatments (Placebo vs Probiotic) for the top 10 families of *P. verrucosa* in T3**	
**Family**	**p-value**
Burkholderiaceae	0.78
Endozoicomonadaceae	0.0068
Lachnospiraceae	**1.80E-05**
Phycisphaeraceae	0.5
Prevotellaceae	**3.10E-05**
Rhodobacteraceae	**0.00042**
Rikenellaceae	**0.00014**
Simkaniaceae	0.077
Unclassified Alphaproteobacteria	0.15
Spirochaetaceae	0.68

Supplementary Table 3. Top 20 most significant differentially abundant ASVs (enriched and decreased) in probiotic treated corals, in comparison to the control, respectively. The ANCOMBC-2 results and the associated taxonomy of the ASVs are given.

Decreased (neg) or enriched (pos)	taxon	new_lab	W statistic	Log Fold Change treatment	q-val treatment	Differentially abundant	Domain	Phylum	Class	Order	Family	Genus
Decreased	ASV_3871	ASV_3871 Anaerobacter	-6.295369	-0.3307025	6.37E-08	TRUE	Bacteria	Firmicutes	Clostridia	Clostridiales	Clostridiaceae	Anaerobacter
Decreased	ASV_33865	ASV_33865 Catenococcus	-8.183557	-0.398387	3.81E-13	TRUE	Bacteria	Proteobacteria	Gammaproteobacteria	Enterobacterales	Vibrionaceae	Catenococcus
Decreased	ASV_15153	ASV_15153 Endozoicomonas	-7.507059	-0.4212	5.02E-11	TRUE	Bacteria	Proteobacteria	Gammaproteobacteria	Pseudomonadales	Endozoicomonadaceae	Endozoicomonas
Decreased	ASV_29809	ASV_29809 Endozoicomonas	-6.592893	-0.3746482	1.79E-08	TRUE	Bacteria	Proteobacteria	Gammaproteobacteria	Pseudomonadales	Endozoicomonadaceae	Endozoicomonas
Decreased	ASV_535	ASV_535 Endozoicomonas	-6.470282	-0.5947397	3.38E-08	TRUE	Bacteria	Proteobacteria	Gammaproteobacteria	Pseudomonadales	Endozoicomonadaceae	Endozoicomonas
Decreased	ASV_520	ASV_520 Endozoicomonas	-6.44588	-0.6574317	3.67E-08	TRUE	Bacteria	Proteobacteria	Gammaproteobacteria	Pseudomonadales	Endozoicomonadaceae	Endozoicomonas
Decreased	ASV_37509	ASV_37509 MND1	-6.295369	-0.3307025	6.37E-08	TRUE	Bacteria	Proteobacteria	Gammaproteobacteria	Burkholderiales	Nitrosomonadaceae	MND1
Decreased	ASV_49869	ASV_49869 MND1	-6.295369	-0.3307025	6.37E-08	TRUE	Bacteria	Proteobacteria	Gammaproteobacteria	Burkholderiales	Nitrosomonadaceae	MND1
Decreased	ASV_19815	ASV_19815 Nitrospira	-6.592893	-0.3746482	1.79E-08	TRUE	Bacteria	Nitrospirota	Nitrospiria	Nitrospirales	Nitrospiraceae	Nitrospira
Decreased	ASV_45942	ASV_45942 Pedobacter	-8.183557	-0.398387	3.81E-13	TRUE	Bacteria	Bacteroidota	Bacteroidia	Sphingobacteriales	Sphingobacteriaceae	Pedobacter
Decreased	ASV_29709	ASV_29709 Povalibacter	-6.295369	-0.3307025	6.37E-08	TRUE	Bacteria	Proteobacteria	Gammaproteobacteria	Steroidobacterales	Steroidobacteraceae	Povalibacter
Decreased	ASV_1208	ASV_1208 Tropicimonas	-6.749979	-0.4538753	1.02E-08	TRUE	Bacteria	Proteobacteria	Alphaproteobacteria	Rhodobacterales	Rhodobacteraceae	Tropicimonas
Decreased	ASV_5717	ASV_5717 Unclassified	-8.4311	-0.4043359	1.42E-13	TRUE	Bacteria	Unclassified	Unclassified	Unclassified	Unclassified	Unclassified
Decreased	ASV_33	ASV_33 Unclassified	-8.140175	-0.5648519	4.10E-13	TRUE	Bacteria	Proteobacteria	Alphaproteobacteria	Puniceispirillales	SAR116 clade	Unclassified
Decreased	ASV_12610	ASV_12610 Unclassified	-6.592893	-0.3746482	1.79E-08	TRUE	Bacteria	Unclassified	Unclassified	Unclassified	Unclassified	Unclassified
Decreased	ASV_45776	ASV_45776 Unclassified	-6.592893	-0.3746482	1.79E-08	TRUE	Bacteria	Gemmatimonadota	Gemmatimonadetes	Gemmatimonadales	Gemmatimonadaceae	Unclassified
Decreased	ASV_15608	ASV_15608 Unclassified	-6.477496	-0.4096574	3.38E-08	TRUE	Bacteria	Proteobacteria	Gammaproteobacteria	Pseudomonadales	P13-46	Unclassified
Decreased	ASV_407	ASV_407 Unclassified	-6.295369	-0.3307025	6.37E-08	TRUE	Bacteria	Proteobacteria	Alphaproteobacteria	Puniceispirillales	SAR116 clade	Unclassified
Decreased	ASV_29808	ASV_29808 Unclassified	-6.295369	-0.3307025	6.37E-08	TRUE	Bacteria	Proteobacteria	Gammaproteobacteria	Pseudomonadales	Unclassified	Unclassified
Decreased	ASV_50372	ASV_50372 Unclassified	-6.295369	-0.3307025	6.37E-08	TRUE	Bacteria	Actinobacteriota	MB-A2-108	Unclassified	Unclassified	Unclassified
Enriched	ASV_1007	ASV_1007 Alcaligenes	4.771578	1.1351577	8.34E-05	TRUE	Bacteria	Proteobacteria	Gammaproteobacteria	Burkholderiales	Alcaligenaceae	Alcaligenes
Enriched	ASV_353	ASV_353 Christensenellaceae R-7 group	5.602933	2.2336347	1.72E-06	TRUE	Bacteria	Firmicutes	Clostridia	Christensenellales	Christensenellaceae	Christensenellaceae R-7 group
Enriched	ASV_567	ASV_567 Delftia	4.984527	1.3280932	3.25E-05	TRUE	Bacteria	Proteobacteria	Gammaproteobacteria	Burkholderiales	Comamonadaceae	Delftia
Enriched	ASV_1666	ASV_1666 Endozoicomonas	5.375482	1.2164077	5.20E-06	TRUE	Bacteria	Proteobacteria	Gammaproteobacteria	Pseudomonadales	Endozoicomonadaceae	Endozoicomonas
Enriched	ASV_2781	ASV_2781 Endozoicomonas	5.039106	0.9874697	2.77E-05	TRUE	Bacteria	Proteobacteria	Gammaproteobacteria	Pseudomonadales	Endozoicomonadaceae	Endozoicomonas
Enriched	ASV_2870	ASV_2870 Endozoicomonas	5.011183	0.9336418	3.12E-05	TRUE	Bacteria	Proteobacteria	Gammaproteobacteria	Pseudomonadales	Endozoicomonadaceae	Endozoicomonas
Enriched	ASV_2179	ASV_2179 Endozoicomonas	4.939459	1.0787632	3.92E-05	TRUE	Bacteria	Proteobacteria	Gammaproteobacteria	Pseudomonadales	Endozoicomonadaceae	Endozoicomonas
Enriched	ASV_634	ASV_634 FD2005	5.15991	1.9236438	1.58E-05	TRUE	Bacteria	Firmicutes	Clostridia	Lachnospirales	Lachnospiraceae	FD2005
Enriched	ASV_1565	ASV_1565 MND1	4.759613	1.5305598	8.66E-05	TRUE	Bacteria	Proteobacteria	Gammaproteobacteria	Burkholderiales	Nitrosomonadaceae	MND1
Enriched	ASV_2683	ASV_2683 Nitrospira	5.447908	1.4802656	3.65E-06	TRUE	Bacteria	Nitrospirota	Nitrospiria	Nitrospirales	Nitrospiraceae	Nitrospira
Enriched	ASV_3443	ASV_3443 Pedomicrobium	4.94728	1.3744393	3.81E-05	TRUE	Bacteria	Proteobacteria	Alphaproteobacteria	Rhizobiales	Hyphomicrobiaceae	Pedomicrobium
Enriched	ASV_1155	ASV_1155 Pseudobutyrivibrio	5.729064	1.8264212	1.05E-06	TRUE	Bacteria	Firmicutes	Clostridia	Lachnospirales	Lachnospiraceae	Pseudobutyrivibrio
Enriched	ASV_2342	ASV_2342 Rikenellaceae RC9 gut group	5.220266	1.5108414	1.16E-05	TRUE	Bacteria	Bacteroidota	Bacteroidia	Bacteroidales	Rikenellaceae	Rikenellaceae RC9 gut group
Enriched	ASV_1211	ASV_1211 Saccharofermentans	5.278231	1.6650278	8.60E-06	TRUE	Bacteria	Firmicutes	Clostridia	Oscillospirales	Hungateiclostridiaceae	Saccharofermentans
Enriched	ASV_208	ASV_208 Salinarimonas	6.27239	2.6235569	7.03E-08	TRUE	Bacteria	Proteobacteria	Alphaproteobacteria	Rhizobiales	Beijerinckiaceae	Salinarimonas
Enriched	ASV_4183	ASV_4183 Simkania	6.166836	1.1394489	1.26E-07	TRUE	Bacteria	Verrucomicrobiota	Chlamydiae	Chlamydiales	Simkaniaceae	Simkania
Enriched	ASV_2431	ASV_2431 Unclassified	5.753954	1.3848494	9.28E-07	TRUE	Bacteria	Proteobacteria	Alphaproteobacteria	Azospirillales	Unclassified	Unclassified
Enriched	ASV_3886	ASV_3886 Unclassified	5.407242	1.3289988	4.50E-06	TRUE	Bacteria	Proteobacteria	Gammaproteobacteria	PLTA13	Unclassified	Unclassified
Enriched	ASV_1840	ASV_1840 Unclassified	4.868957	1.5824654	5.29E-05	TRUE	Bacteria	Bacteroidota	Bacteroidia	Bacteroidales	F082	Unclassified
Enriched	ASV_1089	ASV_1089 Unclassified	4.768441	1.7733261	8.38E-05	TRUE	Bacteria	NB1-j	Unclassified	Unclassified	Unclassified	Unclassified

Supplementary Table 4. Details of the linear mixed effects model analysis for the effect of treatment on the change in *Fv/Fm* monitored at different sampling times. The results of the model selection procedure on the fixed effect terms are given and the most parsimonious model is highlighted in bold. Analyses reveal that treatment was not significant in the analysis. The degrees of freedom (d.f.), AIC, BIC, logLik, Chi square (Chisq) and p-value are given.

**Model selection**
model_3: PAM ~ Time + 1 + (1 | Treatment/Replicate)
model_2: PAM ~ Time + Treatment + (1 | Treatment/Replicate)
model_1: PAM ~ Time * Treatment + (1 | Treatment/Replicate)

	**AIC**	**BIC**	**logLik**	**deviance**	**Chisq**	**Df**	**Pr(>Chisq)**
**model_3**	**-488.856**	**-469.344**	**251.4281**	**-502.856**			
model_2	-486.86	-464.561	251.4302	-502.86	0.00421	1	0.948265
model_1	-483.349	-452.687	252.6747	-505.349	2.489036	3	0.477276

Supplementary Table 5. Details of the post hoc Tukey test comparing the coral in situ performance at different sampling times.

**Sampling time**	**estimate**	**SE**	**df**	**t.ratio**	**p.value**
T1 - T2	-0.03707	0.007301	93.13964	-5.07718	**<.0001**
T1 - T3	-0.08353	0.007301	93.13964	-11.4419	**<.0001**
T1 - T4	-0.01167	0.007301	93.13964	-1.59804	0.38
T2 - T3	-0.04647	0.007301	93.13964	-6.36474	**<.0001**
T2 - T4	0.0254	0.007301	93.13964	3.479149	**0.0042**
T3 - T4	0.071867	0.007301	93.13964	9.843893	**<.00010**

Supplementary Table 6. Details of the post hoc Tukey test comparing the coral thermal threshold (ED50) at different sampling times.

Sampling Time	estimate	SE	df	t.ratio	p.value
T1 - T2	1.479247	0.208432	93.10345	7.097014	**<.0001**
T1 - T3	1.700461	0.208432	93.10345	8.158334	**<.0001**
T1 - T4	2.582236	0.208432	93.10345	12.38884	**<.0001**
T2 - T3	0.221214	0.208432	93.10345	1.06132	0.71
T2 - T4	1.102988	0.208432	93.10345	5.291829	**<.0001**
T3 - T4	0.881775	0.208432	93.10345	4.230508	**<.0001**

Supplementary Table 7. Details of the linear mixed effects model analysis for the effect of treatment on the change in the thermal threshold of *Pocillopora verrucosa* (ED50) monitored at different sampling times. The results of the model selection procedure on the fixed effect terms are given and the most parsimonious model is highlighted in bold. Analyses reveal that treatment was not significant in the analysis. The degrees of freedom (d.f.), AIC, BIC, logLik, Chi square (Chisq) and p-value are given.

Model selection
model_3: ED50 ~ SamplingTimes + 1 + (1 | Site/Replicate)
model_2: ED50 ~ SamplingTimes + Site + (1 | Site/Replicate)
model_1: ED50 ~ SamplingTimes * Site + (1 | Site/Replicate)

	AIC	BIC	logLik	deviance	Chisq	Df	Pr(>Chisq)
**model_3**	**310.5663**	**330.0787**	**-148.283**	**296.5663**			
model_2	312.5083	334.8082	-148.254	296.5083	0.058002	1	0.809683
model_1	317.8613	348.5237	-147.931	295.8613	0.64702	3	0.885589

Supplementary Table 8. *In situ* physicochemical parameters according to sampling time. Daily mean, minimum (Min), and maximum (Max) seawater temperature, daily mean salinity and average nutrient (Silica, Nitrite, Nitrate, Phosphate and Dissolved Organic Carbon [DOC]) concentration are given. Nutrient values were measured in T1 and T3.

Sampling time	date	Water temperature (Min – Max)(°C)	Salinity (PSU)	Silica (µg/l)	Nitrite (µg/l)	Nitrate (µg/l)	Phosphate (µg/l)	DOC (µM/l)
T1	24 August 2021	31.97 (31.64 – 32.39)	39.01	43.21 ± 3.00	6.81 ± 1.80	45.83 ± 30.72	8.84 ± 6.02	126.14 ± 14.88
T2	06 October 2021	29.84 (29.58 – 30.18)	39.14	N/A	N/A	N/A	N/A	N/A
T3	23 November 2021	28.72 (28.54 – 29.01)	38.81	58.17 ± 1.02	16.10 ± 0.69	123.22 ± 82.22	8.15 ± 1.07	171.87 ± 10.58
T4	18 April 2022	28.01 (27.69 – 28.28)	38.91	N/A	N/A	N/A	N/A	N/A

Supplementary Table 9. Temperature profiles and time settings of CBASS experiments.

30°C profile

30°C
18 hrs

33°C profile

	+ 0.6°C						- 0.7°C				
30°C	30.6°C	31.2°C	31.8°C	32.4°C	33°C	33°C	32.3°C	31.6°C	30.9°C	30.2°C	30°C
30 mins	40 mins	40 mins	40 mins	40 mins	20 mins	180 mins	15 mins	15 mins	15 mins	15 mins	660 mins

36°C profile

	+ 1.2°C						- 1.5°C				
30°C	31.2°C	32.4°C	33.6°C	34.8°C	36°C	36°C	34.5°C	33°C	31.5°C	30°C	30°C
30 mins	40 mins	40 mins	40 mins	40 mins	20 mins	180 mins	15 mins	15 mins	15 mins	15 mins	660 mins

39°C profile

	+ 1.8°C						- 2.2°C				
30°C	31.8°C	33.6°C	35.4°C	37.2°C	39°C	39°C	36.8°C	34.6°C	32.4°C	30.2°C	30°C
30 mins	40 mins	40 mins	40 mins	40 mins	20 mins	180 mins	15 mins	15 mins	15 mins	15 mins	660 mins

Acclimatization	Ramping	Heat hold	Cool down	Recovery
30 mins	3 hrs	3 hrs	1 hrs	11 hrs

Supplementary Table 10. ASVs in the coral microbiome that match 100% with the inoculated strains.

ASV	Domain	Phylum	Class	Order	Family	Genus	% identity
ASV_668	Bacteria	Proteobacteria	Gammaproteobacteria	Pseudomonadales	Halomonadaceae	*Cobetia*	100
ASV_836	Bacteria	Proteobacteria	Gammaproteobacteria	Pseudomonadales	Halomonadaceae	*Halomonas*	100
ASV_1662	Bacteria	Proteobacteria	Gammaproteobacteria	Pseudomonadales	Halomonadaceae	*Cobetia*	100
ASV_91250	Bacteria	Proteobacteria	Gammaproteobacteria	Pseudomonadales	Halomonadaceae	*Halomonas*	100
ASV_49725	Bacteria	Proteobacteria	Gammaproteobacteria	Pseudomonadales	Halomonadaceae	*Halomonas*	100
ASV_121537	Bacteria	Proteobacteria	Gammaproteobacteria	Enterobacterales	Pseudoalteromonadaceae	*Pseudoalteromonas*	100
ASV_176301	Bacteria	Proteobacteria	Gammaproteobacteria	Pseudomonadales	Halomonadaceae	*Cobetia*	100
