# Supplementary material for: Probiotics reshape the coral microbiome in situ without detectable off-target effects in the surrounding environment
Source: Commun Biol. 2024 Apr 9;7:434. doi: 10.1038/s42003-024-06135-3 (PMC11004148; doi:10.1038/s42003-024-06135-3)
Supplement: Supplementary file 3 — Reporting summary [file 42003_2024_6135_MOESM3_ESM.pdf]

nature portfolio

Corresponding author(s): Raquel Peixoto

Last updated by author(s): 2024/03/10

Reporting Summary

Nature Portfolio wishes to improve the reproducibility of the work that we publish. This form provides structure for consistency and transparency in reporting. For further information on Nature Portfolio policies, see our Editorial Policies and the Editorial Policy Checklist.

Statistics

For all statistical analyses, confirm that the following items are present in the figure legend, table legend, main text, or Methods section.

n/a	Confirmed	
☐	☒	The exact sample size (*n*) for each experimental group/condition, given as a discrete number and unit of measurement
☐	☒	A statement on whether measurements were taken from distinct samples or whether the same sample was measured repeatedly
☐	☒	The statistical test(s) used AND whether they are one- or two-sided<br>*Only common tests should be described solely by name; describe more complex techniques in the Methods section.*
☐	☒	A description of all covariates tested
☐	☒	A description of any assumptions or corrections, such as tests of normality and adjustment for multiple comparisons
☐	☒	A full description of the statistical parameters including central tendency (e.g. means) or other basic estimates (e.g. regression coefficient) AND variation (e.g. standard deviation) or associated estimates of uncertainty (e.g. confidence intervals)
☐	☒	For null hypothesis testing, the test statistic (e.g. *F*, *t*, *r*) with confidence intervals, effect sizes, degrees of freedom and *P* value noted<br>*Give P values as exact values whenever suitable.*
☒	☐	For Bayesian analysis, information on the choice of priors and Markov chain Monte Carlo settings
☐	☒	For hierarchical and complex designs, identification of the appropriate level for tests and full reporting of outcomes
☐	☒	Estimates of effect sizes (e.g. Cohen's *d*, Pearson's *r*), indicating how they were calculated

Our web collection on statistics for biologists contains articles on many of the points above.

Software and code

Policy information about availability of computer code

Data collection	All datasets were generated from original data from the field experiment conducted. Original raw 16S rRNA amplicon sequences from the coral, seawater and sediment datasets are available in the European Nucleotide Archive (ENA) under the study accession number PRJEB65896. Other data supporting the results of this study are provided as Supplementary data files. Large data sets are available in Zenodo DOI: 10.5281/zenodo.10801800.
Data analysis	Coral, seawater and sediment datasets were analyzed in R studio (R v 4.2.2) using the functions in Phyloseq version 1.42.0 124 and Vegan version 2.6-4 125. All plots were generated using ggplot2 version 3.4.0.The costume code is available in the Zenodo repository DOI: 10.5281/zenodo.10801800

For manuscripts utilizing custom algorithms or software that are central to the research but not yet described in published literature, software must be made available to editors and reviewers. We strongly encourage code deposition in a community repository (e.g. GitHub). See the Nature Portfolio guidelines for submitting code & software for further information.

Data

Policy information about availability of data

All manuscripts must include a data availability statement. This statement should provide the following information, where applicable:

- Accession codes, unique identifiers, or web links for publicly available datasets
- A description of any restrictions on data availability
- For clinical datasets or third party data, please ensure that the statement adheres to our policy

All sequence reads were deposited in the European Nucleotide Archive (ENA) under the study accession number PRJEB65896. Large data sets required to replicate and generate the results of this study are available on Zenodo DOI: 10.5281/zenodo.10801800.

Research involving human participants, their data, or biological material

Policy information about studies with human participants or human data. See also policy information about sex, gender (identity/presentation), and sexual orientation and race, ethnicity and racism.

Reporting on sex and gender	N/A
Reporting on race, ethnicity, or other socially relevant groupings	N/A
Population characteristics	N/A
Recruitment	N/A
Ethics oversight	N/A

Note that full information on the approval of the study protocol must also be provided in the manuscript.

Field-specific reporting

Please select the one below that is the best fit for your research. If you are not sure, read the appropriate sections before making your selection.

☐ Life sciences ☐ Behavioural & social sciences ☒ Ecological, evolutionary & environmental sciences

For a reference copy of the document with all sections, see nature.com/documents/nr-reporting-summary-flat.pdf

Ecological, evolutionary & environmental sciences study design

All studies must disclose on these points even when the disclosure is negative.

Study description	An experiment consisting of assembling a bacterial probiotic consortium (consisting of six strains) isolated from healthy corals in the Red Sea and its in situ application in healthy colonies of Pocillopora verrucosa in a mid-shore reef in the Central Red Sea, to test its effect on the coral microbiome and monitor the surrounding microbiomes of seawater and sediment, as well as the coral physiology in response to the probiotic treatment.
Research sample	Experimental data consisting of 15 healthy colonies of Pocillopora verrrucosa per treatment, (placebo and probiotic) evaluated during 4 sampling times. Seawater and sediment samples (n = 5 per treatment, respectively) were collected at two sampling times (T1 & T3).
Sampling strategy	The same coral colonies were tagged permanently and sampled during 4 sampling times, covering seasonal variations (T1-T4) from late August, 2021, until early April, 2022. The colonies were selected based on their visual health status and randomly assigned to the treatments using a random number generator. The colonies were 3 meters apart from each other to avoid clonal genotypes.
Data collection	15 colonies per treatment used for the study were sampled for bacterial community and physiology analyses. Seawater and sediment samples (n= 5 per treatment, respectively) were collected at T1 & T3 sampling times for bacterial community analyses.
Timing and spatial scale	All data was recorded between August 2021, and April, 2022.
Data exclusions	No data exclusions were done for the current study. Six samples from corals (C_CTRL9T1, C_CTRL4_T3, C_BMC8T1, C_BMC13T2, C_BMC14T2 and C_BMC2T4) did not pass the quality control for the sequencing, so they were not included in the analyses.
Reproducibility	Reproducibility of all analyses is guarantee by using the provided available R codes and datasets.
Randomization	Randomization was used to assign treatments (placebo and probiotic) to the coral colonies (biological replicates) in the study area.

Randomization	Seawater and sediment samples near the coral colonies were collected from random coral colonies using a random number generator.
Blinding	No blinding was implemented in this study.

Did the study involve field work? ☒ Yes ☐ No

Field work, collection and transport

Field conditions	Calm to slightly wavy sea conditions. The depth ranged from 8 -10 m. The study area corresponds to a non-exposed site of the reef.
Location	The study site was located in a shallow sheltered area (8 - 9 m depth) in "Al Fahal Reef" (22°18'18.4"N; 38°57'52.5"E), a mid-shore reef in the central Red Sea, 15 km off-shore from King Abdullah University of Science and Technology (KAUST), Saudi Arabia.
Access & import/export	All data collection was taken under the local Coastguard authorization and the university authorization to sample biological material in nearby reefs.
Disturbance	Disturbance was minimum to collect small fragments (4-5 cm in diameter) of coral, 2L of seawater and 50 g of sediment nearby the studied coral colonies, in the different sampling times. All procedures were carried out by Scuba diving, with minimum disturbance in the reef environment.

Reporting for specific materials, systems and methods

We require information from authors about some types of materials, experimental systems and methods used in many studies. Here, indicate whether each material, system or method listed is relevant to your study. If you are not sure if a list item applies to your research, read the appropriate section before selecting a response.

Materials & experimental systems

n/a	Involved in the study	
☒	☐	Antibodies
☒	☐	Eukaryotic cell lines
☒	☐	Palaeontology and archaeology
☐	☒	Animals and other organisms
☒	☐	Clinical data
☒	☐	Dual use research of concern
☒	☐	Plants

Methods

n/a	Involved in the study	
☒	☐	ChIP-seq
☒	☐	Flow cytometry
☒	☐	MRI-based neuroimaging

Animals and other research organisms

Policy information about studies involving animals; ARRIVE guidelines recommended for reporting animal research, and Sex and Gender in Research

Laboratory animals	N/A
Wild animals	This study used coral colonies of Pocillopora verrucosa in situ.
Reporting on sex	N/A
Field-collected samples	Coral fragments, seawater and sediment samples were collected from the studied coral colonies of Pocillopora verrucosa and their surrounding environment. A total of 120 coral samples were collected (30 samples per sampling time, 15 per treatment) in T1-T4, 10 samples of seawater and 10 samples of sediment (5 per reatment) in T1 & T3. Samples were transported to the laboratory less than 3 hours after collection, and preserved in liquid nitrogen with DESS buffer (coral samples) and ice (water and sediment samples). Coral and sediment samples were maintained at -80 Celsius degrees until processing. Water samples were filtered immediately in the laboratory.
Ethics oversight	No ethic approvals were required for this study.

Note that full information on the approval of the study protocol must also be provided in the manuscript.

Plants

Seed stocks	N/A
Novel plant genotypes	N/A
Authentication	N/A
